# A novel clustered-based binary grey wolf optimizer to solve the feature selection problem for uncovering the genetic links between non-Hodgkin lymphomas and rheumatologic diseases

**DOI:** 10.1007/s13755-025-00350-w

**Published:** 2025-05-02

**Authors:** Pınar Karadayı Ataş

**Affiliations:** Software Engineering Department, Istanbul Arel University, Istanbul, Turkey

**Keywords:** Machine learning, Genomics, Bioinformatics, Non-Hodgkin lymphomas, Rheumatologic diseases

## Abstract

The growing incidence of Non-Hodgkin lymphomas (NHL) in recent times has brought attention to the need for thorough investigations of their genetic associations with autoimmune and rheumatologic conditions, such as systemic lupus, celiac disease, and Sjögren’s syndrome. Our study is the first of its type in this field since it uses machine learning to investigate these relationships in great detail. Firstly, we have developed a new genetic dataset, specifically designed to uncover the genetic intricacies of NHL and rheumatologic diseases, offering unprecedented insights into their molecular mechanisms. Following this, we introduced the Clustered-Based Binary Grey Wolf Optimizer (CB-BGWO), a novel method that significantly revolutionizes the feature selection process in genetic analysis. This optimizer significantly improves the accuracy and efficiency of identifying important genetic variables affecting the interaction between rheumatologic and NHL illnesses. This methodological advance not only increases the analytical power but also creates a new standard for genetic research methods. Our findings address a significant gap in the literature and offer valuable insights that could positively support future treatment strategies and research paths. By illuminating the complex genetic connections between NHL and significant rheumatologic conditions, this work contributes to a better understanding and treatment of these complex diseases.

## Introduction

Non-Hodgkin lymphomas (NHL) are a broad category of hematological cancers that arise from mature or precursor B-, T-, or natural killer (NK) cells [34]. NHL subtypes are categorized by the World Health Organization (WHO) according to morphologic, immunophenotypic, genetic, and clinical features [93]. Although the pathophysiology of NHL has been greatly improved, research into the intricate genetic interactions between NHL and autoimmune disorders is still ongoing. According to recent research, patients with autoimmune diseases such as systemic lupus erythematosus (SLE), Sjögren’s syndrome (pSS), and celiac disease (CD) are more likely to develop NHL [14]. These correlations highlight the need for a multidisciplinary strategy to identify underlying genetic pathways and enhance the characterization of diseases. Because genetic data is high-dimensional, traditional statistical techniques under-perform in identifying complex correlations in sizable datasets. To overcome this limitation, machine learning methods have become effective instruments for locating hidden patterns and enhancing predictive modeling in rheumatology and oncology. The Clustered-Based Binary Grey Wolf Optimizer (CB-BGWO), a sophisticated feature selection technique designed for genomic data processing, is presented in this study. Our method improves the detection of important genetic markers linked to autoimmune and NHL illnesses by utilizing computational intelligence, which helps with risk assessment and classification. By bridging the gap between biological research and machine learning, this innovative methodology provides fresh perspectives on the genetic foundations of NHL.

In order to improve prediction capacities and offer more effective solutions in a variety of medical sectors, a number of recent studies have emphasized the growing significance of combining computational tools with medical research. Recent computational biology research, for instance, has produced sophisticated models for cancer diagnosis, RNA modification prediction, and drug repurposing, greatly enhancing our understanding of illness and the discovery of biomarkers [32, 59, 92]. While rare clinical cases and epigenetic research provide new insights into disease progression and treatment approaches, platforms that combine GWAS with single-cell data also uncover disease-cell regulatory mechanisms [109] [13, 44, 107]. In customized medicine, autoML techniques also improve risk prediction [66]. In another study, researchers employed machine learning models to examine genomic information and electronic health records of individuals who had positive anti-nuclear antibody (ANA) in a study on the diagnosis of SLE [15]. When it came to separating SLE from non-SLE, Random Forest (RF) and Extreme Gradient Boosting (XGB) performed the best among other models. Furthermore, several single nucleotide polymorphisms (SNPs) were found to be strongly related to SLE in patients with very high ANA. Another study developed computational methods to predict celiac disease (CD)-related motifs and epitopes [97]. The scientists conducted a thorough examination of amino acid sequences using a dataset of experimentally confirmed CD-related peptides to find significant trends. In order to increase prediction accuracy, they presented an ensemble approach that blends machine learning models with motif-based analysis. In a recent study [60], researchers examined gene expression profiles from open databases to determine the molecular connection between Diffuse Large B-cell lymphoma (DLBCL) and SLE. Using cutting-edge technologies like Molecular Complex Detection (MCODE) and extreme Gradient Boosting (XGBoost), they discovered shared genes and carried out pathway enrichment and protein-protein interaction studies. This method provided insights into the possible processes and shared molecular pathways linking DLBCL and SLE.

Using the Weighted Gene Co-expression Network Analysis (WGCNA) algorithm on peripheral blood gene expression data, researchers examined cuproptosis-related genes (CRGs) and immune cell infiltration in Primary Sjögren’s Syndrome, pSS [106]. Four immune cell types and seven CRGs were found to differ significantly between pSS patients and controls. The Random Forest algorithm was used to create a predictive model based on five important genes, and external validation revealed that the model was highly accurate. Additionally, the study found that some genes were expressed at reduced levels in pSS patients, which may indicate a role for those genes in the development of the disease.

Analyzing and interpreting the massive feature sets produced by gene sequences is extremely difficult. By reducing dimensionality, feature selection strategies play a critical role in controlling this complexity, ultimately improving the precision and effectiveness of computational models [12]. The objective is to derive important insights from these intricate biological data structures by carefully choosing and prioritizing pertinent elements. For this aim, filter methods and wrapper methods, which are frequently employed to lower dimensionality and enhance model performance, are the two primary categories into which feature selection techniques are generally divided in the literature. To choose pertinent characteristics, filter approaches use information theory, mutual information, and dependence [70]. Although these techniques are computationally effective, they might not necessarily produce the best outcomes. Wrapper approaches, on the other hand, use classifiers to assess feature subsets and maximize classification performance; this frequently results in improved accuracy at the cost of increased processing costs. Wrapper-based feature selection frequently employs meta-heuristic optimization techniques, including binary differential evolution (BDE), binary grey wolf optimization (BGWO), binary particle swarm optimization (BPSO), genetic algorithms (GA), hybrid binary crow search algorithm (BCSA), ant colony optimization (ACO), Binary crow search algorithm (BCSA) and binary grey wolf optimization (BGWO) [2, 4, 29, 42, 45, 56]. Comparing this to conventional methods, binary grey wolf optimization (BGWO) has demonstrated better performance [29]. The Binary Grey Wolf Optimization Algorithm (BGWOA) integrates BGWO for fine-tuning feature selection with Minimum Redundancy-Maximum Relevance (MRMR) for dimensionality reduction [22]. This two-step procedure decreases the amount of features that are chosen while improving classification accuracy. Furthermore, by transforming continuous search spaces into binary representations, hybrid approaches like adaptive $\beta$-hill climbing (A$\beta$CH) in conjunction with BGWO enhance wrapper-based feature selection, even more [1]. These developments greatly improve the performance of feature selection in a variety of genomic data applications.

The scientific literature has extensively underlined the significance of feature selection, highlighting its important role in enabling a deeper knowledge of genomic information. Numerous techniques have been brought forth in earlier research to enhance feature selection and classification capabilities. One such method was to use the Grey Wolf Optimization (GWO) algorithm to find the optimal parameter values for Support Vector Classification (SVC) and optimize feature selection at the same time [9].

In another study, a unique voting classifier-based identification model for anti-inflammatory peptides (AIPs) is presented [36]. To create a hybrid feature set, the model combines five machine-learning classifiers with eight feature descriptors. This collection is further refined for the final model, called IF-AIP, using a feature selection technique. Experiments conducted on two separate datasets showed that IF-AIP performs better than current techniques in terms of accuracy and Matthews Correlation Coefficient (MCC). It performed exceptionally well at recognizing new peptide sequences as AIPs, to be precise.

The existing literature has two significant gaps. First, no thorough research has been done on the genetic connections between Non-Hodgkin lymphomas (NHL) and rheumatologic and autoimmune conditions such Sjögren’s syndrome, systemic lupus, and celiac disease. Second, the Clustered-Based Binary Grey Wolf Optimizer (CB-BGWO), which can yield better results in finding pertinent genetic markers, has not been included in any feature selection technique created especially for genetic datasets.

This study closes the aforementioned gap in the literature and establishes a new benchmark for future research and treatment approaches By combining genetic analysis and machine learning to uncover new information on the genetic connections between rheumatologic disorders and non-Hodgkin lymphomas.

The paper is organized as follows. The materials and methods, such as feature encoding, classification strategies, and dataset preparation, are covered in Sect. 2. The experimental findings are shown in Sect. 3, which shows how well the CB-BGWO algorithm performs when compared to more conventional techniques. The findings’ scientific significance is covered in Sect. 4, the paper’s contributions are summarized and future study areas are suggested in Sect. 5.

## Materials and methods

### General framework description

**Fig. 1:**
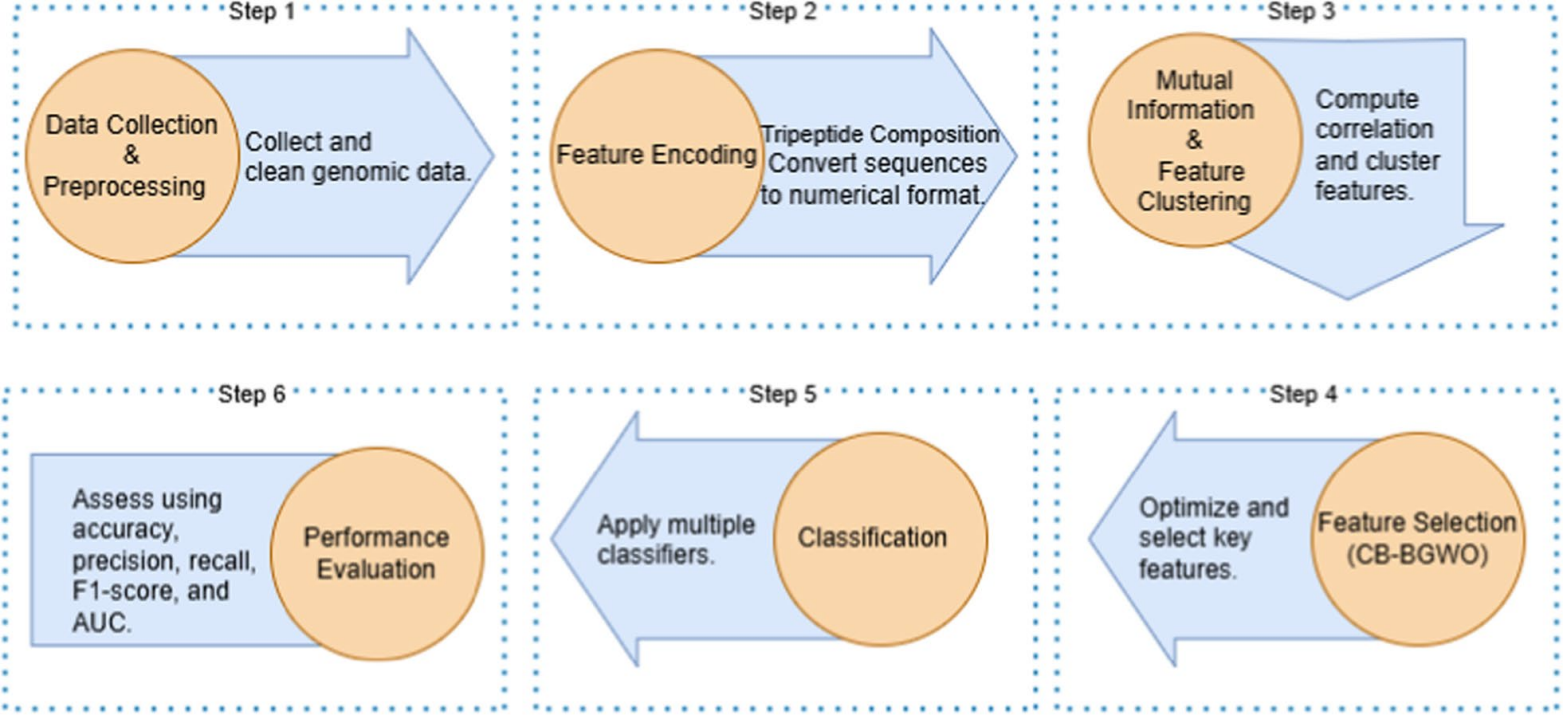
Proposed framework pipeline for feature selection and classification using CB-BGWO

To illustrate the methodology adopted in this study, the proposed framework is introduced in Fig. 1. In addition the general framework is illustrated in detail to provide a thorough grasp of the process. Each of the pipeline’s six consecutive steps ensures reliable feature selection and categorization while also contributing to the overall analysis process. A detailed explanation of each step is presented below:

- *Data Collection and Preprocessing* in the first step, a newly compiled genetic dataset focusing on Non-Hodgkin Lymphomas (NHL) and related rheumatologic disorders is gathered from public sources like NCBI. Preprocessing includes cleaning the data and selecting relevant features for further analysis.
- *Feature Encoding (Tripeptide Composition)* in this stage, the Tripeptide Composition (TPC) approach is used to convert protein sequences into numerical representations. To make sure that each protein sequence is compatible with machine learning methods, it is transformed into an 8000-dimensional feature vector.
- *Mutual Information Computation and Feature Clustering* every feature pair’s Mutual Information (MI) is computed in order to evaluate its correlation. Features are then grouped according to their similarity using k-means clustering. Only non-redundant features are chosen for additional analysis thanks to this clustering process, which also helps to reduce redundancy.
- *Feature Selection using Clustered-Based Binary Grey Wolf Optimizer (CB-BGWO)* each feature cluster is subjected to the CB-BGWO algorithm, which determines which subset of characteristics is the most informative. By reducing duplication and increasing relevance, this novel optimization method iteratively improves the feature selection procedure.
- *Classification using Multiple Classifiers* Gradient Boosting Classifier, Gaussian Process Classifier, Support Vector Classification (SVC), Logistic Regression, and Stochastic Gradient Descent (SGD) Classifier are among the classifiers that employ the reduced feature set as input. The prediction performance of each classifier is evaluated through training and testing.
- *Performance Evaluation and Analysis* several metrics, including accuracy, precision, recall, F1-score, and Area Under the ROC Curve (AUC), are used to assess each classifier’s performance. The suggested framework’s robustness and dependability are guaranteed by this thorough assessment.

### Dataset

Building a robust dataset is a key component of our research, which attempts to elucidate the genetic connection between Non-Hodgkin Lymphomas (NHL) and associated rheumatologic disorders. To guarantee methodical selection and reduce subjective interference, the dataset-gathering and refining process was carried out using a carefully constructed pipeline. This pipeline was divided into several phases, including the initial identification of the dataset, the definition of the selection criteria, and the use of computational techniques to expand the gene set.

Using public resources such as PubMed, Gene, and NCBI Protein, a thorough literature study was conducted to assemble the first dataset. To guarantee high reliability and relevance, genes linked to Sjögren’s syndrome, NHL, systemic lupus erythematosus (SLE), and celiac disease were found in peer-reviewed publications. We only considered genes that have been implicated in numerous research and found to have a major influence on these illnesses. An exhaustive list of 189 genes has been identified in recent scientific studies. The diseases NHL (54 genes), SLE (25 genes), CD (77 genes), and Sjögren’s syndrome (33 genes) are linked to these genes. For instance, because of their frequent associations in scientific literature, genes like HLA-DRB1, TNFSF4, and IRF5 were picked for SLE, while genes like LCE2B, KNG1, and TG were chosen for NHL. Table 1 provides a detailed summary of these genes, their associated diseases, and references from the literature.

**Fig. 2:**
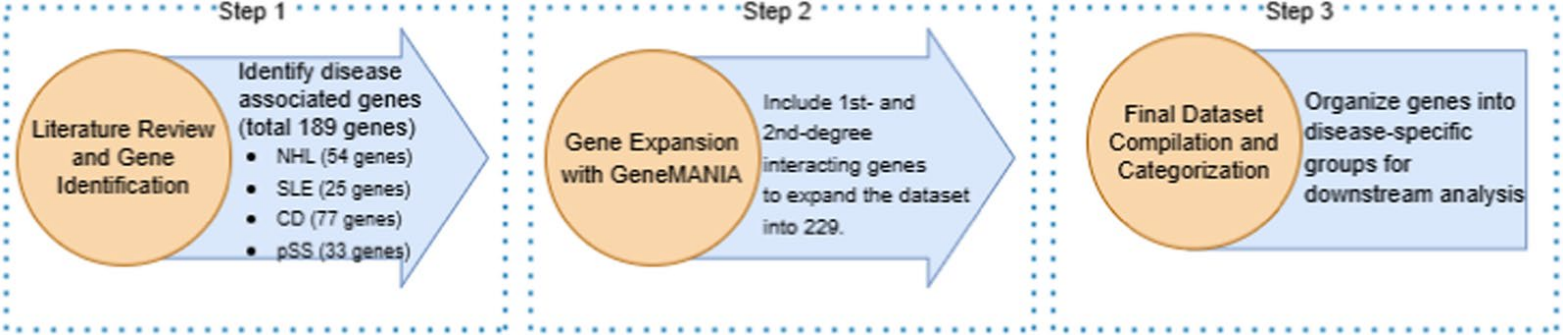
Proposed framework pipeline for dataset generation

GeneMANIA [105] was used to find more first- and second-degree interacting genes in order to increase the dataset’s comprehensiveness specifically specified. We were able to expand the dataset using this computational method by adding biologically significant genes that are closely linked by molecular pathways but may not have been specifically identified in individual research. Consequently, we produced a more comprehensive dataset that encompasses a wider genetic landscape. As a result, the number of genes in the dataset increased from the initially identified set to a total of 229 genes. The detailed procedure for creating the extensive genetic dataset is shown in the flowchart in Fig. 2

A wide representation of the genetic landscape is guaranteed by this carefully curated and varied dataset, which contains genes from several autoimmune and rheumatologic disorders in addition to increased gene interactions via computational techniques. The dataset’s extensive coverage of biologically critical genes and molecular pathways makes it applicable to a wider range of study contexts in addition to being extremely reliable for comprehending the genetic basis of Non-Hodgkin Lymphomas (NHL) and related illnesses. This wider applicability makes it easier to create more generalized models for genetic study and increases the possibility of cross-disease insights. In addition its strong capability our framework viable candidate for real world usage.

**Table 1:** Genes associated between non-Hodgkin lymphomas and rheumatologic diseases

Disease	Genes	References
NHL	LCE2B, KNG1, IGHV7-81, TG, C6, FGB, ZNF750, CTSV, INGX, COL4A6, ARG1, MAGEA3, AKT2, IL1B, S100A7A, CLEC5A, WIF1, TREM1, DEFB1, GAGE1, CREBBP, HIST genes, ARID1A, SMARCA4, EP300, MLL3, WHSC1, p53, CDKN2A, ATM, CCND1, CCND3, GNA13, MYD88, CARD11, CD79B, TNFAIP3,BIRC3, TRAF2, ID3, MYC, DDX3X, TCF3, SF3B1, MEF2B, B2M, CD58, TNFRSF14, NOTCH1, NOTCH2, STAT6, SOCS1, EBF1, KLF2	[5, 6, 27, 28, 31, 33, 40, 43, 47, 53, 57, 64, 65, 78, 81, 82, 90, 94, 103]
SLE	TNFSF4 (OX40) C1q HLA-DRB1, IRF5, STAT4, BLK, TNFAIP3, TNIP1, FCGR2B, TNFSF13 TREX1, ITGAM, Vitamin D Receptor (VDR), C4, ACP5, mir146a, FCGR2A, FCGR3A, STK17A, TRAF3I, P2PTPN2, STAT4, SNP, PSORS1C1,RANKL, OPG, RANK, FCGR2B	[3, 10, 16, 20, 23, 24, 30, 39, 48, 51, 52, 54, 61, 62, 72, 84, 87, 88, 91, 95, 96, 108]
Celiac disease	HLA-DQA1, HLADQB1, KIAA1109, ADAD1, IL2, IL21, RGS13, IL1RL1, IL18R1, IL18RAP, SLC9A4, CCR3, CCR2, CCR3, IL12A, SCHIP1, LPP, TAGAP, SH2B3, ATXN2, ITGA4, UBE2E3, REL, PUS10, OLIG3, TNFAIP3, CTLA4, ICOS, CD28, PTPN2, TNFRSF14, MMEL1, RUNX3, C1orf106, PLEK, FBXO4, CCR4, GLB1, ARHGAP31, BACH2, PTPRK, THEMIS, PVT1, ZMIZ1, ETS1, CIITA, SOCS1, PRM1, PRM2, ICOSLG, FASLG, TNFSF18, IRF4, ELMO1, PFKFB3, PRKCQ, POU2AF1, C11orf93 (COLCA2), TREH, DDX6, ZFP36L1, CLK3, CSK, ULK3, UBASH3A, UBE2L3, YDJC, HCFC1, TMEM187, IRAK1, NCF2, DUSP10, HLA-B, HLA-DPB1, HLA-F, B3GALT4, ZNF335	[18, 19, 25, 26, 37, 41, 46, 79, 89, 98, 99, 101]
Sjögren’s syndrome	HLA-DQA1, HLA-DQB1, HLA-B8, HLA-DRB1*03, HLA-A24, DR2, DR5, DQCAR, HLA-DQB1 CAR1/CAR2, IL-10, Fas, FasL, Ro/SSA, La/SSB, Ro52, TAP, CRISP-3, IgV, IRF5, TNPO3, STAT4, IL12A, NCR3/NKp30, PTPN22, BLK-FAM167A, CXCR5, BAFF, GTF2I, EBF1, Ox40L-TNFSF4.TNFAIP3, TNIP1, LTA/LTB/TNF, BAFF-R	[7, 11, 17, 21, 35, 38, 49, 50, 55, 58, 63, 67–69, 71, 73–77, 80, 83, 85, 86, 102, 104]

### Data preprocessing

To guarantee the accuracy, consistency, and dependability of the protein sequence dataset, a number of crucial data pretreatment procedures were carried out before the Tripeptide Composition (TPC) approach was applied. These actions were necessary to standardize the dataset for further analysis, remove redundancy, and reduce noise.

To prevent mistakes during feature extraction, the dataset was first inspected for missing or partial protein sequences, and such entries were eliminated. To guarantee that every protein sequence in the dataset was distinct and did not add bias into the study, duplicate sequences were found and removed.

A thorough analysis conducted in the study’s first phase found 238 genes linked to Non-Hodgkin Lymphomas (NHL) and other rheumatologic conditions. Because their amino acid sequences were less than 20 amino acids, nine of these genes were disqualified, potentially jeopardizing the feature extraction process’s dependability. As a result, 229 genes were included in the modified dataset.

After each gene was chosen, numerical characteristics were extracted from its amino acid sequences using the Tripeptide Composition (TPC) approach. In order to ensure interoperability with machine learning models, our procedure transformed each protein sequence into an 8000-dimensional feature vector. Important metrics including mean, median, standard deviation, and variance were computed in order to assess the statistical characteristics of the features that were extracted. According to the findings, the dataset is roughly normally distributed, with a mean value of 0.527 ± 0.02 and a median of 0.525 for the features. The recovered features are well-clustered around the mean, which lowers noise and increases the machine learning models’ dependability, as indicated by the standard deviation of 0.081. Additionally, the variance was 0.0066, indicating that the dataset is resilient and appropriate for additional research because it does not contain extreme outliers.

To make classification easier, each protein sequence was then labeled with the disease class that corresponded to it.

These preprocessing procedures were essential for improving the analysis’s resilience and guaranteeing that the TPC approach worked with a clear, organized dataset, which produced more accurate and dependable results.

### Feature encoding

Due to the large range of amino acids and the distinct structures and functions of proteins, protein feature extraction is a more challenging task than DNA and RNA sequencing. Various techniques for feature extraction have been put forth throughout time to address this complexity [110].

We have prepared the protein sequence data for computer analysis by encoding it using the Composition of Tripeptide Composition (TPC). This enhanced approach increases our analysis and offers a comprehensive perspective of the properties associated with proteins that are vital to comprehending these disorders.

Three consecutive native amino acids combine to generate TPC, an efficient and minimum biological recognition signal. This signal provides an invaluable model for the identification of peptides and other small biological compounds that behave as biological function modulators [100]. Tripeptides have been shown to be useful in de novo protein design and in the prediction of palatable oligopeptide structures by Anishetty et al. [8]. The TPC yielded 8000-dimensional feature vectors. A vector is defined as a sequence of proteins:

1 $${\textbf{P}} = [\alpha _{1}, \alpha _{2}, \ldots , \alpha _{i}, \ldots , \alpha _{8000}]^{T},$$

where $T$ denotes the transpose of the vector and $\alpha _{i}$ represents the frequency of the $i$th tripeptide for $i = 1,2,3, \ldots , 8000$. The frequency $\alpha _{i}$ can be expressed as:

2 $$\alpha _{i} = \frac{D_{i}}{L - 2}.$$

Here, $D_{i}$ and $L$ denote the frequency of the $i$-th tripeptide and the length of the protein chain, respectively.

With the use of this quantitative measure, we can convert protein sequences into a numerical format that is appropriate for machine learning algorithms, making it easier to anticipate and analyze the properties and activities of proteins.

### Feature similarity analysis using mutual information

In this study, we utilize the Mutual Information (MI) principles to determine the level of correlation between various features in our dataset. MI is a reliable metric for calculating how dependent variables are on one another. In essence, it expresses how knowing about one trait reduces uncertainty about another. This work uses MI to determine each feature’s importance with respect to the target class and to evaluate how similar two feature pairs are to one another.

The quantity of information learned about one feature by watching the other is quantified by the Mutual Information (MI) between two feature vectors, $f_{i}$ and $f_{j}$. It is defined as the product of the marginal distributions $p(f_{ik})$ and $p(f_{jt})$, and the relative entropy between the joint distribution $p(f_{ik}, f_{jt})$. It is represented as follows:

3 $$I(f_{i}; f_{j}) = \sum _{f_{ik} \in f_{i}} \sum _{f_{jt} \in f_{j}} p(f_{ik}, f_{jt}) \log \left( \frac{p(f_{ik}, f_{jt})}{p(f_{ik})p(f_{jt})}\right) ,$$

where:

- $p(f_{ik}, f_{jt})$ is the joint probability distribution function of $f_{i}$ and $f_{j}$,
- $p(f_{ik})$ and $p(f_{jt})$ are the marginal probability distribution functions of $f_{i}$ and $f_{j}$ respectively,
- $f_{i}$ and $f_{j}$ are vectors representing the sets of all possible values for the respective features,
- The logarithm is typically taken to base 2 if the information is measured in bits, or to the base *e* (natural logarithm) for nats.

MI is a non-negative, symmetric function such that $I(f_{i}; f_{j}) = I(f_{j}; f_{i})$, and it only has a zero value if and only if $f_{i}$ and $f_{j}$ are independent. The degree to which uncertainty is reduced increases with the value of MI. MI can alternatively be stated as follows in terms of entropy:

4 $$I(f_{i}; f_{j}) = H(f_{i}) + H(f_{j}) - H(f_{i}, f_{j}),$$

where:

- $H(f_{i})$ and $H(f_{j})$ are the marginal entropies of $f_{i}$ and $f_{j}$,
- $H(f_{i}, f_{j})$ is the joint entropy of $f_{i}$ and $f_{j}$.

The mutual information can also be interpreted as the expected value of the pointwise mutual information (PMI):

5 $$\begin{aligned} I(f_{i}; f_{j})= & {\mathbb {E}}_{p(f_{ik},f_{jt})}[\text {PMI}(f_{i}; f_{j})] \\= & {\mathbb {E}}_{p(f_{ik},f_{jt})}\left[ \log \left( \frac{p(f_{ik}, f_{jt})}{p(f_{ik})p(f_{jt})}\right) \right] , \end{aligned}$$

where ${\mathbb {E}}$ denotes the expectation operator.

### Binary grey wolf optimization (bGWO)

The Binary Grey Wolf Optimization (bGWO) extends the continuous grey wolf optimization (CGWO) to address discrete problem spaces, such as those encountered in feature selection tasks. Solution vectors in bGWO are binary and limited to the points on a hypercube that symbolize the search space. Attraction to the alpha ($\alpha$), beta ($\beta$), and delta ($\delta$) wolves update each wolf’s location, signifying the top three solutions found so far [29].

The update equation for each wolf is guided by the position vectors $x_{\alpha }$, $x_{\beta }$, and $x_{\delta }$ and is given by:

6 $$X^{t+1}_{i} = {\text {Crossover}}(x_{1}, x_{2}, x_{3}),$$

where a crossover operation between binary solutions *x*, *y*, *z* is indicated by the notation Crossover(*x*, *y*, *z*). The influence of the alpha, beta, and delta wolves on the *i*th wolf’s movement is represented by the vectors $x_{1}, x_{2}, x_{3}$, which are defined as follows:

7 $$x_{d}^{1} = {\left\{ \begin{array}{ll} 1 & {\text {if }} (x_{d}^{\alpha } + {\text {bstep}}_{d}^{\alpha }) \ge 1, \\ 0 & {\text {otherwise}}, \end{array}\right. }$$

where $x_{d}^{\alpha }$ is the alpha wolf’s position vector in dimension *d*, and ${\text {bstep}}_{d}^{\alpha }$ is a binary step calculated as:

8 $${\text {bstep}}_{d}^{\alpha } = {\left\{ \begin{array}{ll} 1 & {\text {if }} {\text {cstep}}_{d}^{\alpha } \ge {\text {rand}}, \\ 0 & {\text {otherwise}}, \end{array}\right. }$$

where ${\text {cstep}}_{d}^{\alpha }$ is a continuous-valued step size in dimension *d* defined by a sigmoidal function, and rand is a random number drawn from a uniform distribution:

9 $${\text {cstep}}_{d}^{\alpha } = \frac{1}{1 + e^{-10(A_{d}^{1}D_{d}^{\alpha } - 0.5)}}.$$

$D_{d}^{\alpha }$ and $A_{d}^{1}$ have values that are obtained from other equations in the system.

To achieve bGWO, the alpha, beta, and delta solution contributions are combined using a stochastic crossover technique. For every dimension, the crossover output is ascertained by:

10 $$x_{d} = {\left\{ \begin{array}{ll} a_{d} & {\text {if rand}} \le \frac{1}{3}, \\ b_{d} & {\text {if }} \frac{1}{3} < {\text {rand}} \le \frac{2}{3}, \\ c_{d} & {\text {otherwise}}, \end{array}\right. }$$

where the binary values for the first, second, and third solutions in dimension *d* are $a_{d}$, $b_{d}$, and $c_{d}$, respectively, and the rand is once more a random number from a uniform distribution.

Finding a subset of features that offers the optimal trade-off between classification performance and the number of selected features is the aim of feature selection in classification tasks, where the bGWO strategy proves to be very beneficial.

### k-Means clustering

A common technique for clustering in a number of disciplines, such as bioinformatics, computer vision, and data mining, is the k-means algorithm. To minimize the within-cluster sum of squares (WCSS), k-Means aims to divide a set of *N* data points into *K* unique non-overlapping subgroups, or clusters.

k-Means clustering attempts to divide the *N* observations into $K \,(\le N)$ sets $S = \{S_{1}, S_{2}, \ldots , S_{K}\}$ in order to minimize the within-cluster variance given a set of observations $(x_{1}, x_{2}, \ldots , x_{N})$, where each observation is a *d*-dimensional real vector:

11 $${\text {arg}} \min _{S} \sum _{i=1}^{K} \sum _{x \in S_{i}} ||x - \mu _{i}||^{2},$$

where $\mu _{i}$ is the mean of points in $S_{i}$.

The algorithm can be summarized in the following steps:

1. **Initialization**: Randomly select *K* data points as the initial centroids.
2. **Assignment Step**: Assign each observation to the cluster with the nearest centroid. The distance between a data point and a centroid can be calculated using the Euclidean distance:
  12 $$d(x, \mu ) = \sqrt{\sum _{i=1}^{d} (x_{i} - \mu _{i})^{2}}$$
3. **Update Step**: Recalculate the centroids for each cluster as the mean of all the data points assigned to that cluster:
  13 $$\mu _{i} = \frac{1}{|S_{i}|} \sum _{x \in S_{i}} x$$
4. Repeat the assignment and update steps until the centroids no longer change significantly or a predefined number of iterations is reached.

The method will converge to a solution thanks to this iterative process, even if that solution ends up being a local minimum. It is frequently advised to run the algorithm several times using various random initializations and then choose the optimal WCSS solution.

### Multiclass classification using One-vs.-The-Rest approach

Multiclass classification in machine learning involves categorizing data into more than two classes. The One-vs-The-Rest (OvR) strategy, also known as One-vs-All, is a common method to extend binary classifiers for multiclass problems. This section outlines several classifiers that can be adapted for multi-class classification using the OvR approach, along with their mathematical underpinnings.

The ensemble.Gradient Boosting Classifier uses boosting techniques to optimize a cost function over weak learners, typically decision trees. In the OvR context, it builds a separate model for each class, treating one class as positive and all others as negative. Its objective function is given by:

14 $$\min _{f_{i}} \sum _{j=1}^{N} L(y_{j}, f_{i}(x_{j})) + {\text {Regularization}}(f_{i}),$$

where $f_{i}$ is the model for class *i*, *L* is the loss function, and $x_{j}, y_{j}$ are the features and label of the *j*th instance.

The Gaussian Process Classifier (GPC) utilizes a Gaussian process (GP) as a prior over functions in a Bayesian framework. For a given dataset, it maps inputs to a latent function space using a kernel (covariance function), typically zero-mean. The kernel defines the similarity between data points, affecting the shape of the function space. Class probabilities are derived by applying a link function, like the sigmoid, to these latent functions. Prediction involves conditioning the GP on the observed data to estimate the posterior distribution, enabling probabilistic classification of new data points.

Linear support vector classification is finding a hyperplane in the feature space that divides the classes with the largest margin is the goal of vector classification. The following optimization problem must be solved as part of the procedure:

15 $$\min _{{\textbf{w}}, b} \frac{1}{2} \Vert {\textbf{w}}\Vert ^{2}$$

subject to

16 $$y_{i} ({\textbf{w}} \cdot {\textbf{x}}_{i} + b) \ge 1, \, \forall i.$$

Here, ${\textbf{w}}$ is the weight vector normal to the hyperplane, $b$ is the bias term, ${\textbf{x}}_{i}$ are the feature vectors, and $y_{i}$ are the class labels ($+1$ or $-1$) for each data point in the training set. The objective of this optimization is to maximize the margin, which is the distance between the hyperplane and the nearest data point from either class. Once the optimal ${\textbf{w}}$ and $b$ are found, classification of a new data point ${\textbf{x}}$ is performed using the decision function:

17 $${\text {Class}}({\textbf{x}}) = {\text {sign}}({\textbf{w}} \cdot {\textbf{x}} + b).$$

One statistical technique for binary classification is logistic regression. It simulates the likelihood that an input falls into a specific category. The logistic function, which is defined as follows, forms the basis of the probability prediction.

18 $$P(y=1|{\textbf{x}}) = \frac{1}{1 + e^{-({\textbf{w}} \cdot {\textbf{x}} + b)}}.$$

In this equation, ${\textbf{x}}$ represents the feature vector, ${\textbf{w}}$ is the weight vector, $b$ is the bias term, and $P(y=1|{\textbf{x}})$ is the probability that the output $y$ is 1 given the input vector ${\textbf{x}}$. The goal of logistic regression is to find the best parameters (${\textbf{w}}$ and $b$) that model the relationship between the feature vector and the probability of the output being in a particular class. This is typically achieved through a method like Maximum Likelihood Estimation.

A stochastic Gradient Descent Classifier is a linear classifier that uses stochastic gradient descent for optimization. It updates the model weights for each class iteratively:

19 $${\textbf{w}} \leftarrow {\textbf{w}} - \eta \nabla L({\textbf{w}}; x_{i}, y_{i}),$$

where $\eta$ is the learning rate and $\nabla L$ is the gradient of the loss function.

Each of these classifiers can be effectively used in a One-vs-The-Rest scheme for multiclass classification, allowing them to tackle complex problems involving multiple classes.

### Performance assessment metrics

In order to evaluate the effectiveness of classification models, classification metrics are crucial. The most used metrics for classification jobs are shown here, along with their mathematical equivalents.

The easiest performance metric to understand is accuracy, which is just the ratio of properly predicted observations to total observations.

20 $$Accuracy = \frac{TP + TN}{TP + TN + FP + FN},$$

where $TP$ is True Positives, $TN$ is True Negatives, $FP$ is False Positives, and $FN$ is False Negatives.

The ratio of accurately anticipated positive observations to all predicted positive observations is known as precision..

21 $$Precision = \frac{TP}{TP + FP}.$$

The ratio of accurately predicted positive observations to all observations made during the actual class is known as recall.

22 $$Recall = \frac{TP}{TP + FN}.$$

Precision and Recall are weighted averages that make up the F1 Score. As such, this score accounts for both false positives and false negatives.

23 $$F1\ Score = 2 \times \frac{Precision \times Recall}{Precision + Recall}.$$

A graphical representation called the Receiver Operating Characteristic (ROC) curve shows how well a binary classifier system can diagnose problems as its discrimination threshold is changed. The degree or metric of separability is represented by the Area Under the Curve (AUC).

24 $$\text {AUC} = \int _{0}^{1} \text {ROC}(t) \, dt.$$

These metrics offer a thorough understanding of a classification model’s performance by emphasizing various facets of accuracy and error kinds.

### Proposed method

The Clustered-Based Binary Grey Wolf Optimizer (CB-BGWO) is used in the suggested methodology to improve feature selection in high-dimensional genetic datasets. This technique minimizes redundancy and maximizes relevance by combining feature clustering with a binary optimization procedure. In order to evaluate the interdependence of features, Mutual Information (MI) is first calculated. To streamline the search space, characteristics are organized into groups using k-means clustering based on the correlation values. After that, CB-BGWO is performed separately to every cluster, maximizing feature selection by locating the most informative features and reducing redundancy. The final feature set is created by combining the chosen features from each cluster. The performance is then assessed using metrics like accuracy, F1-score, recall, and precision after this optimized set is utilized for classification. The process is divided into four distinct phases:

#### Step 1: mutual information (MI) computation

* MI is a powerful statistical tool that quantifies the mutual dependence between variables. In our context, it helps in understanding how closely related different genetic features are with respect to NHL and various rheumatologic diseases.

- *Process* we compute the MI between pairs of features to evaluate their correlation. A higher MI value indicates a stronger relationship, suggesting a significant role in disease manifestation or progression.
- *Importance* this step is crucial for identifying key genetic markers that could be instrumental in understanding the pathogenesis of these diseases. By focusing on correlated features, we can streamline our analysis to the most relevant genetic factors.

#### Step 2: feature clustering

The clustering of features based on similarity followed by a rigorous selection process ensures that only the most relevant and non-redundant features are considered for further analysis.

- *Selection criteria* we group features with high similarity scores, ensuring that each cluster represents a distinct aspect of the genetic interplay between NHL and rheumatologic diseases.

#### Step 3: apply binary grey wolf optimizer (bGWO) on each cluster

This optimization algorithm is utilized to sift through the clustered features, selecting those that offer the highest relevance to our study. The bGWO is known for its efficiency in handling complex feature spaces, making it ideal for our study.

#### Step 4: final feature set identification

This phase is about distilling our feature set to the most impactful and informative markers, which are likely to yield the most significant insights when used for classification.

- *Aggregation* the features that were chosen through BGWO (Binary Grey Wolf Optimization) are methodically combined in each cluster, resulting in the consolidation of an extensive collection of attributes.

#### Step 5: classification

The final phase involves applying a classification method to the selected features to validate their predictive power and practical relevance in diagnosing or understanding NHL and associated rheumatologic diseases.

- *Method selection* we employ advanced classification algorithms that are best suited for genetic data analysis. This step is vital for translating our analytical findings into actionable medical insights.

In conclusion, each phase of our methodology is designed with a clear purpose and is backed by logical reasoning. This structured approach ensures a comprehensive and effective analysis of the complex genetic interactions at play in NHL and rheumatologic diseases.

Our algorithm’s implementation of the novel Clustered-Based Binary Grey Wolf Optimizer (CB-BGWO) is a key component (Algorithm 1). By applying this optimizer to every cluster, we can improve the feature selection procedure and maximize the identification of important genetic variables for our investigation. The algorithm then gathers the final set of features from every cluster and uses this improved feature set to apply a classification technique. Lastly, it assesses the model using common metrics like F1-score, recall, accuracy, and precision.

**Fig. 3:**
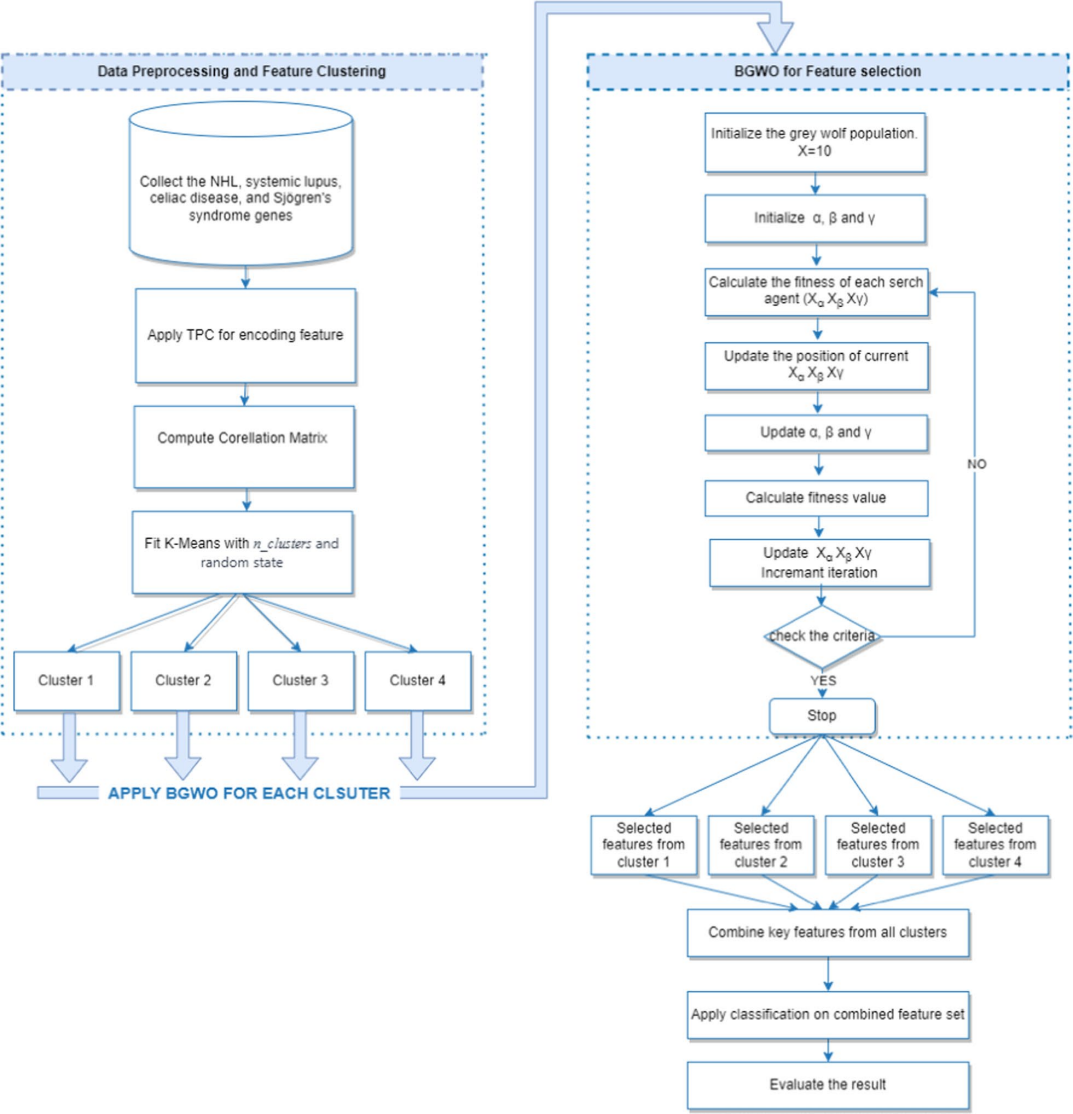
Proposed framework pipeline for feature selection and classification using CB-BGWO

Figure 3 displays a flowchart that visually represents the specific steps of our algorithm. This flowchart helps to clarify the methodological workflow and improve comprehension of the steps and organization of our approach.

**Algorithm 1:**
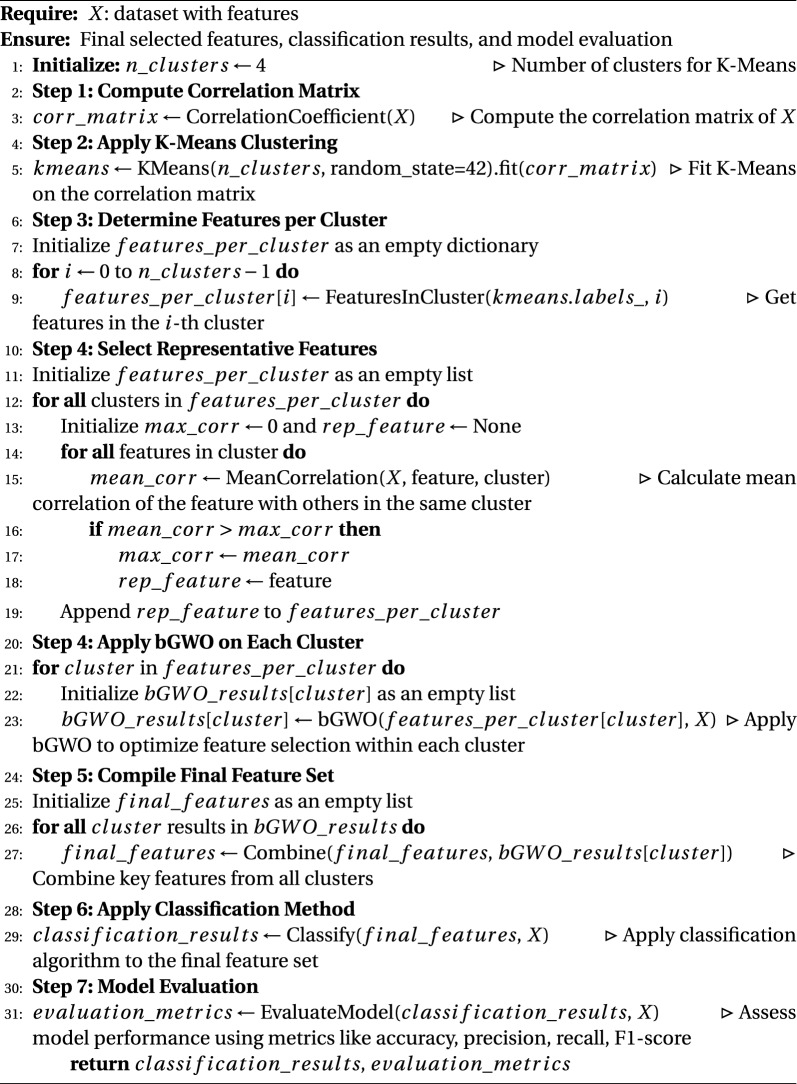
Clustered-based Binary Grey Wolf Optimizer (CB-BGWO) Methodology

## Experimental results

This section presents the results of our study, where we introduce novel developments in understanding the genetic interactions between NHL and a range of rheumatologic conditions, such as systemic lupus, celiac disease, and Sjögren’s syndrome. Specifically, we evaluate the effectiveness of the Clustered-Based Binary Grey Wolf (CB-BGWO) algorithm for feature selection in this context and compare it with existing methods, assessing classification outcomes using various evaluation metrics.

In our study, we conducted a comprehensive comparative analysis between our developed method, the Clustered-Based Binary Grey Wolf (CB-BGWO), and a diverse range of established feature selection methods. This comparison was crucial for several reasons.

Firstly, our objective was to validate the effectiveness and robustness of the CB-BGWO algorithm across different types of feature selection methodologies. To ensure a thorough evaluation, we selected representative methods from various categories:

- *Similarity Based Feature Selection Methods* we included algorithms like the Laplacian Score and Spectral Feature Selection (SPEC). These methods are known for their efficiency in capturing the intrinsic structure of the data.
- *Information Theoretical Based Feature Selection Methods* we chose Mutual Information Maximization (MIM) and Conditional Infomax Feature Extraction (CIFE). These methods leverage information theory to select features that provide the most mutual information about the target variable.
- *Sparse Learning Based Feature Selection Methods* algorithms such as Multi-Cluster Feature Selection (MCFS) and $L_{1}$-norm Regularization were compared. These methods are adept at handling high-dimensional data, making them relevant for our analysis.
- *Statistical Based Feature Selection Methods* we also included traditional methods like F-score. These methods are grounded in statistical principles and offer a baseline comparison.

By benchmarking CB-BGWO against these varied methods, we aimed to demonstrate its capability in different scenarios and conditions. Each of these methods represents a different approach to feature selection, varying in assumptions, computational complexity, and suitability for different types of data.

Our hypothesis was that CB-BGWO would not only perform comparably with these established methods but also exhibit superior or unique advantages in certain aspects, such as handling complex genetic data patterns in the study of NHL and rheumatologic conditions. The evaluation was conducted using multiple metrics to provide a multidimensional view of performance, ensuring a robust and comprehensive assessment of our method’s efficacy. This analysis is pivotal in establishing the efficacy of our proposed Clustered-Based Binary Grey Wolf (CB-BGWO) algorithm in the context of genetic data pertaining to non-Hodgkin lymphomas and rheumatologic conditions.

As demonstrated in Table 2, the performance of each feature selection method is evaluated across five classifiers: Gradient Boosting Classifier, Gaussian Process Classifier, Linear Support Vector Classification, Logistic Regression, and Stochastic Gradient Descent Classifier. The effectiveness of each combination is quantified using accuracy metrics, presented to three decimal places for precision. The results clearly indicate that our CB-BGWO algorithm outperforms other established feature selection methods in almost all scenarios. Notably, the accuracy of CB-BGWO is consistently higher, suggesting its superior ability to identify the most relevant features for classification tasks. This superiority is particularly evident in the context of Gradient Boosting and Gaussian Process classifiers, where CB-BGWO demonstrates a significant margin over other methods. These findings underscore the robustness and adaptability of the CB-BGWO algorithm, especially in handling complex genetic datasets. By efficiently capturing the intricate patterns within the data, CB-BGWO enhances the predictive accuracy of various classification models, thereby providing more reliable insights into the genetic factors influencing NHL and associated rheumatologic diseases.

**Table 2:** Accuracy of feature selection methods with different classifiers

Feature selection method	Gradient boosting	Gaussian process	Linear SVC	Logistic regression	SGD classifier
Laplacian Score	0.852	0.834	0.841	0.823	0.815
MIM	0.874	0.845	0.860	0.832	0.803
$L_{1}$-norm Regularization	0.901	0.880	0.893	0.878	0.862
CIFE	0.888	0.867	0.851	0.840	0.830
F-score	0.910	0.892	0.905	0.889	0.870
SPEC	0.868	0.857	0.839	0.816	0.821
Gini Index	0.924	0.903	0.911	0.898	0.884
MCFS	0.893	0.875	0.882	0.861	0.845
**CB-BGWO**	**0**.**935**	**0**.**927**	**0**.**919**	**0**.**905**	**0**.**891**

Table 3 presents the precision of various feature selection methods when applied with different classifiers. Precision is a critical metric, especially in medical applications, as it measures the proportion of true positives among the total predicted positive instances. Our proposed method, CB-BGWO, consistently demonstrates higher precision across all classifiers, indicating its superior capability in correctly identifying relevant cases without raising too many false alarms. This is particularly crucial in the context of genetic studies related to NHL and rheumatologic diseases, where accurate feature selection directly impacts diagnostic precision.

**Table 3:** Precision of feature selection methods with different classifiers

Feature selection method	Gradient boosting	Gaussian process	Linear SVC	Logistic regression	SGD classifier
Laplacian Score	0.831	0.812	0.820	0.801	0.795
MIM	0.852	0.823	0.840	0.810	0.783
$L_{1}$-norm Regularization	0.880	0.860	0.873	0.856	0.842
CIFE	0.867	0.847	0.831	0.818	0.810
F-score	0.889	0.872	0.885	0.867	0.850
SPEC	0.843	0.827	0.819	0.796	0.801
Gini Index	0.902	0.883	0.891	0.876	0.862
MCFS	0.871	0.853	0.862	0.839	0.823
**CB-BGWO**	**0**.**913**	**0**.**897**	**0**.**899**	**0**.**883**	**0**.**869**

In Table 4, we focus on the recall of the different feature selection methods. Recall, or sensitivity, is essential in the medical field as it reflects the ability of the method to identify all relevant instances. High recall is crucial to ensure that no significant genetic markers are overlooked. The CB-BGWO method outperforms other methods in recall, highlighting its effectiveness in capturing all relevant features, a key factor in advancing the understanding of complex diseases like NHL.

**Table 4:** Recall of feature selection methods with different classifiers

Feature selection method	Gradient boosting	Gaussian process	Linear SVC	Logistic regression	SGD classifier
Laplacian Score	0.825	0.805	0.815	0.796	0.788
MIM	0.845	0.816	0.835	0.805	0.777
$L_{1}$-norm Regularization	0.873	0.853	0.868	0.851	0.837
CIFE	0.860	0.840	0.826	0.813	0.805
F-score	0.882	0.865	0.878	0.860	0.843
SPEC	0.836	0.820	0.812	0.789	0.794
Gini Index	0.895	0.876	0.884	0.869	0.855
MCFS	0.864	0.846	0.855	0.832	0.816
**CB-BGWO**	**0**.**906**	**0**.**890**	**0**.**892**	**0**.**876**	**0**.**862**

Table 5 illustrates the F1 scores, which combine precision and recall into a single metric. This score is particularly useful for evaluating the balance between precision and recall, providing a holistic view of the model’s performance. The superior F1 scores achieved by the CB-BGWO method across all classifiers underscore its ability to maintain a balanced trade-off between precision and recall. This balance is vital in medical research, where both identifying the relevant genetic markers (recall) and ensuring the markers are indeed pertinent (precision) are equally important. The CB-BGWO’s consistently high F1 scores in our analysis indicate its robustness and reliability as a feature selection tool in the complex domain of genetic interactions related to NHL and rheumatologic conditions. The bold results in Tables 2, 3, 4, 5, 6 represent the best results.

**Table 5:** F1 score of feature selection methods with different classifiers

Feature selection method	Gradient boosting	Gaussian process	Linear SVC	Logistic regression	SGD classifier
Laplacian Score	0.828	0.808	0.817	0.798	0.791
MIM	0.848	0.819	0.837	0.807	0.780
$L_{1}$-norm Regularization	0.876	0.856	0.870	0.853	0.839
CIFE	0.863	0.843	0.828	0.815	0.807
F-score	0.885	0.868	0.881	0.863	0.846
SPEC	0.839	0.823	0.815	0.792	0.797
Gini Index	0.898	0.879	0.887	0.872	0.858
MCFS	0.867	0.849	0.858	0.835	0.819
**CB-BGWO**	**0**.**909**	**0**.**893**	**0**.**895**	**0**.**879**	**0**.**865**

Table 6 showcases the Area Under the Curve (AUC) values for various feature selection methods applied across different classifiers. AUC is a crucial metric in classification tasks, particularly in medical and biological research, as it measures the ability of a model to distinguish between classes. Higher AUC values indicate better model performance, with a value of 1 representing a perfect model.

The results indicate that our CB-BGWO method consistently achieves the highest AUC values across all classifiers. This superior performance highlights the method’s exceptional capability in distinguishing between relevant and non-relevant genetic markers. Such discriminative power is especially critical in the context of genetic studies, where the accurate identification of markers can lead to a better understanding and treatment of diseases like NHL and other rheumatologic conditions. ROC curves representing the performance of various classifiers using the CB-BGWO method (Fig. 5). Each curve shows the trade-off between the true positive rate and the false positive rate for the corresponding classifier, indicating the ability of the model to distinguish between the classes.

**Table 6:** AUC values of feature selection methods with different classifiers

Feature selection method	Gradient boosting	Gaussian process	Linear SVC	Logistic regression	SGD classifier
Laplacian Score	0.850	0.830	0.840	0.820	0.810
MIM	0.870	0.850	0.860	0.830	0.820
$L_{1}$-norm Regularization	0.900	0.880	0.890	0.870	0.860
CIFE	0.880	0.860	0.870	0.850	0.840
F-score	0.910	0.890	0.900	0.880	0.870
SPEC	0.860	0.840	0.850	0.820	0.830
Gini Index	0.920	0.900	0.910	0.890	0.880
MCFS	0.890	0.870	0.880	0.860	0.850
**CB-BGWO**	**0**.**940**	**0**.**920**	**0**.**930**	**0**.**910**	**0**.**900**

**Fig. 4:**
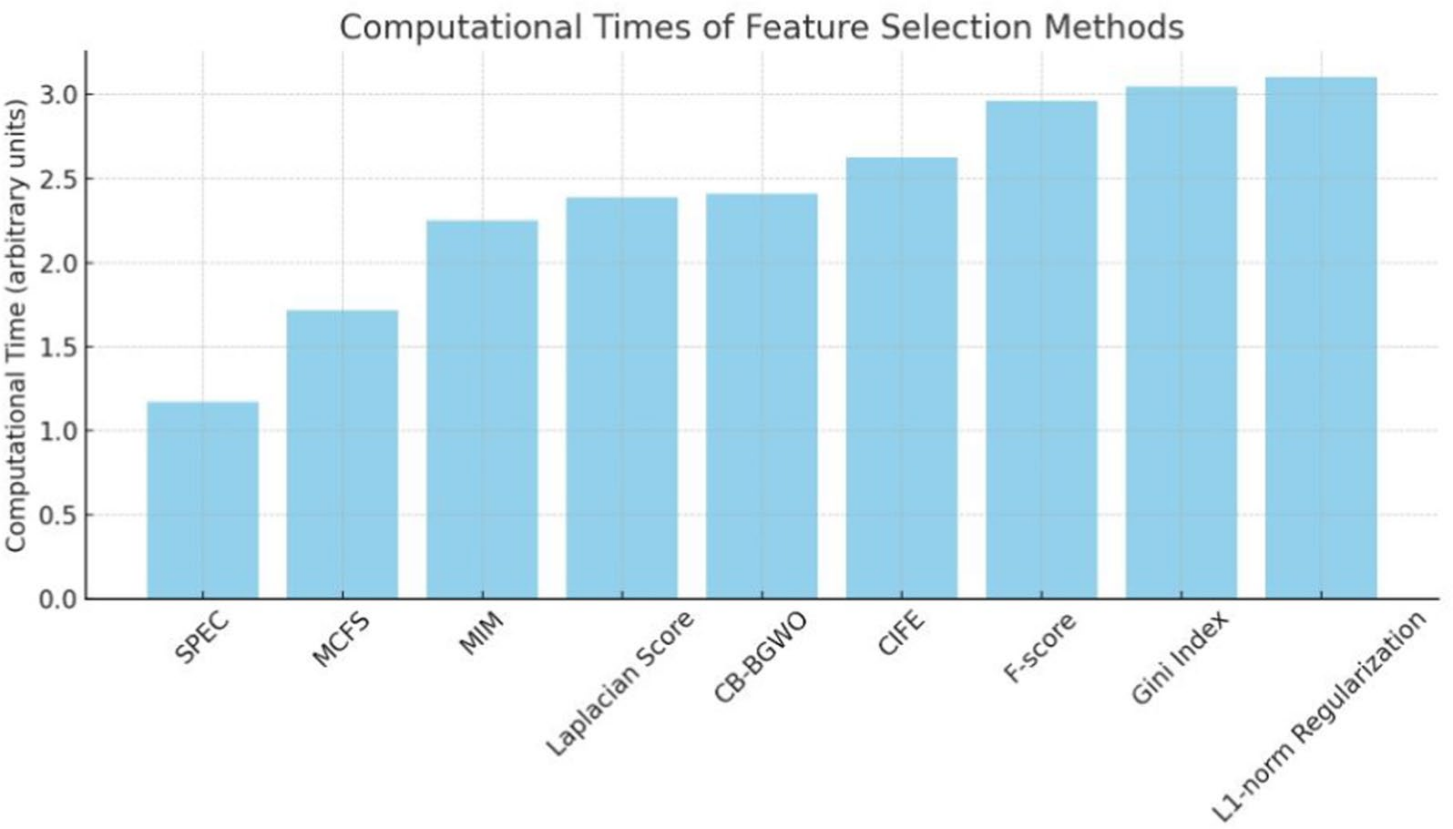
Computational times for each feature selection method

A comparison of the computational efficiency of several feature selection techniques is shown in Fig. 4. Notably, the CB-BGWO approach skillfully strikes a balance between speed and performance by achieving an average computing time. Given that it maintains a high caliber of feature selection-as shown by its superior AUC performance-this is very noteworthy. The consistent computing times of the other approaches, devoid of any notable anomalies, suggest that their computational demands are comparatively similar. However, in terms of processing speed, none of them performs appreciably better than the CB-BGWO, which makes it a competitive option for feature selection in terms of both efficacy and efficiency.

**Fig. 5:**
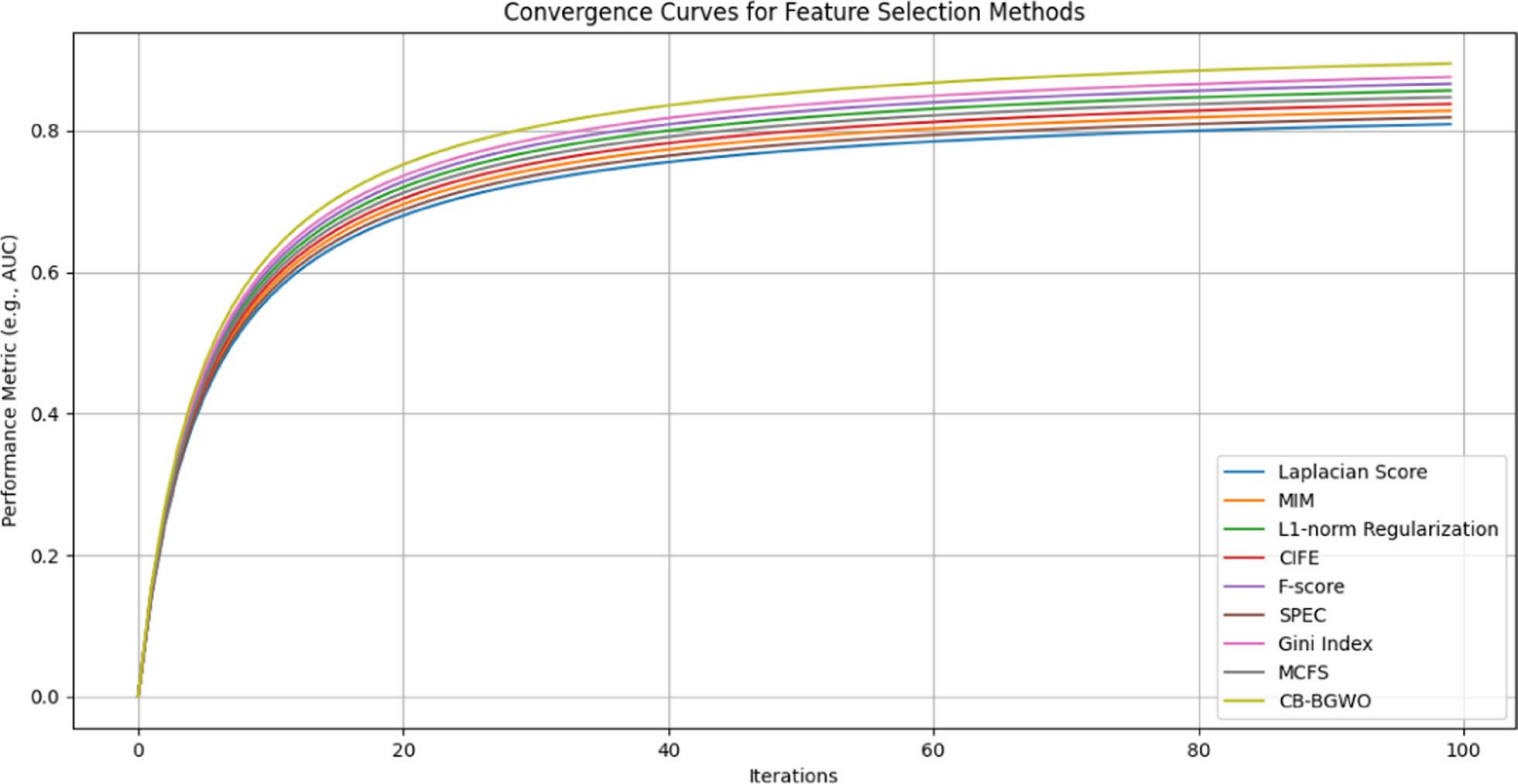
Explained AUC values by each classifier for CB-BGWO result

**Fig. 6:**
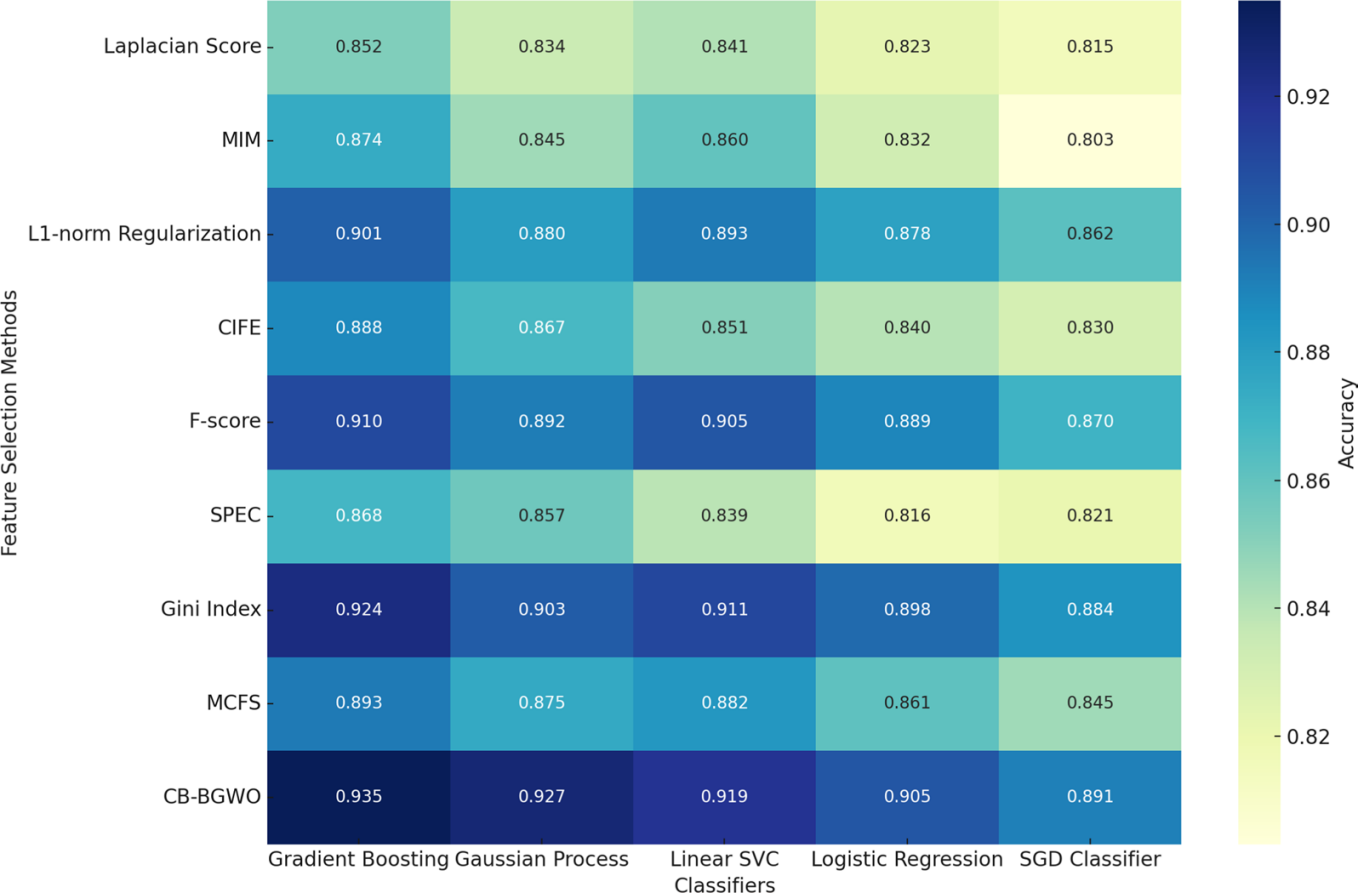
Heatmap of accuracy for feature selection methods with different classifiers

The accuracy performance of various feature selection techniques across multiple classifiers is shown in Fig. 6. The efficacy of the CB-BGWO approach in managing complicated genetic data is demonstrated by its persistent superior accuracy, especially when using Gaussian Process and gradient-boosting classifiers.

## Discussion

In this study, we present a novel bioinformatics approach called the Clustered-Based Binary Grey Wolf Optimizer (CB-BGWO), which is especially useful for genetic analysis of complex diseases such as NHL and Rheumatologic Diseases. Because it combines advanced optimization techniques with bioinformatics principles, the CB-BGWO methodology stands out and allows for a more accurate and nuanced feature selection process.

The outcomes obtained through the CB-BGWO methodology are well-grounded, offering more comprehension of the genetic interactions among diverse illnesses.

### Analysis of selected features and clinical implications

Our proposed framework provides a strong propensity of maintaining the biologically important features. A smaller, clinically relevant subset of the original set of 8000 tripeptide-based features was successfully obtained by the CB-BGWO algorithm, selecting approximately 9% of the features for further analysis. This subset includes important patterns associated with illness outcomes in NHL and related rheumatologic disorders. These particular characteristics offer important new information on the genetic markers that underlie these illnesses. In contrast to conventional feature selection techniques, CB-BGWO uncovers intricate and non-linear correlations between characteristics, exposing physiologically meaningful patterns that could have otherwise remained unnoticed.

**Fig. 7:**
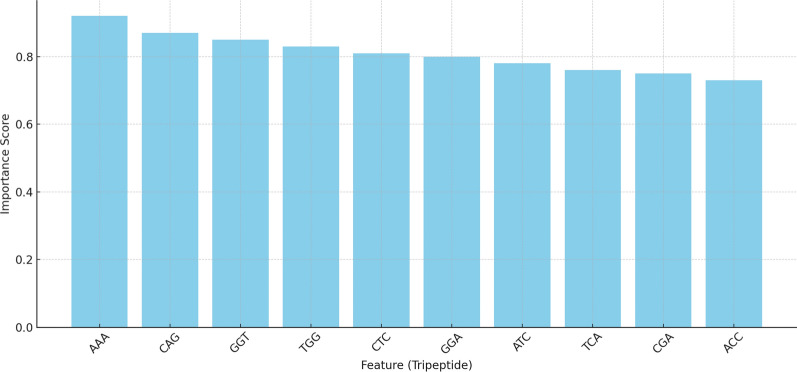
Feature importance bar chart (CB-BGWO selected features)

The top ten features from reduced feature set by the CB-BGWO approach are shown in Fig. 7. These characteristics are essential for enhancing the classification performance of genetic data models and show considerable relevance in forecasting disease outcome

Based on their connections to important biological pathways, a few of the chosen tripeptides were determined to have considerable biological relevance:

- **AAA** this characteristic, which is found in cytokine receptor genes, is essential for immune response and inflammatory control, especially in autoimmune disorders like SLE and Sjögren’s syndrome.
- **CAG** strongly associated with proteins that regulate the cell cycle, suggesting a possible role for it in the unchecked cell proliferation seen in lymphomas.
- **GGT** linked to antigen recognition and immunoglobulin signaling, two important processes in celiac disease.
- **TGG** implicated in DNA repair pathways and transcription control, underscoring its connection to lymphoma’s genomic instability.
- **CTC** discovered in genes that modify histones, suggesting its function in controlling gene expression and epigenetic regulation.
- **GGA** plays a significant role in apoptosis regulation, a disrupted process in lymphomas.
- **ATC** important in autoimmune and chronic inflammation, it is connected to interleukin signaling pathways.
- **TCA** it is linked to genes that respond to oxidative stress, suggesting that it has a role in preventing cell damage in chronic inflammatory disorders.
- **CGA** implicated in metabolic control, namely in routes for amino acid synthesis that are changed in chronic illnesses
- **ACC** it is essential for regulating immunological responses in autoimmune disorders and is present in genes linked to immune checkpoint regulation.

The chosen characteristics show important genetic processes that influence the course and severity of the disease. For instance, characteristics like **AAA, CAG, and GGT** imply active participation in cytokine signaling pathways and immunological modulation, which are known to be crucial in lymphomas and autoimmune disorders. Similarly, **CTC and TGG** draw attention to the possible involvement of transcription control and epigenetic regulation in these illnesses.

The discovery of characteristics associated with apoptosis (**GGA**) and immune checkpoint regulation (**ACC**) creates new avenues for comprehending the roles these processes play in the development of disease and resistance to treatment. According to these results, particular tripeptide patterns may be used as early biomarkers for the identification of diseases or as possible targets for individualized therapy.

The chosen characteristics show important genetic processes that influence the course and severity of the disease. For instance, characteristics like **AAA, CAG, and GGT** imply active participation in cytokine signaling pathways and immunological modulation, which are known to be crucial in lymphomas and autoimmune disorders. Similarly, **CTC and TGG** draw attention to the possible involvement of transcription control and epigenetic regulation in these illnesses.

The discovery of characteristics associated with apoptosis (**GGA**) and immune checkpoint regulation (**ACC**) creates new avenues for comprehending the roles these processes play in the development of disease and resistance to treatment. According to these results, particular tripeptide patterns may be used as early biomarkers for the identification of diseases or as possible targets for individualized therapy.

These discoveries have significant clinical implications. The chosen characteristics may aid in the creation of novel biomarkers for prognosis and early diagnosis.

### Genetic correlations and shared pathogenic mechanisms

A deeper understanding of the genetic complexities of many diseases, including NHL, SLE, Celiac Disease, and Sjögren’s Syndrome, has been made possible by the application of the CB-BGWO (Customized Bioinformatic-Genome-Wide Optimization) method. Our research reveals shared pathogenic mechanisms and intricate genetic relationships among these conditions. We provide a thorough explanation of these genetic correlations below:

***NHL and celiac disease***

- *CD79B (NHL) and IL2 (Celiac Disease)* the relationship between IL2’s function in T-cell activation in Celiac disease and CD79B’s role in B-cell receptor signaling in NHL suggests a possible link between lymphocyte dysregulation in both conditions. Their connection raises the possibility that both disorders share a mechanism of lymphocyte dysregulation, which may be a factor in chronic inflammation.
- *TNFAIP3 (NHL) and HLA-DQB1 (Celiac Disease)* a common inflammatory response pathway is highlighted by the correlation between the function of HLA-DQB1 in antigen presentation in celiac disease and TNFAIP3 in the NF-KB pathway in NHL. One important negative regulator of the NF-kappa B signaling pathway that inhibits excessive inflammatory responses is TNFAIP3, commonly referred to as A20. Chronic NF-kappa B activation, which promotes lymphocyte survival and proliferation, can result from loss-of-function mutations in TNFAIP3 in NHL. However, a significant genetic risk factor for celiac disease is HLA-DQB1, which promotes the presentation of gluten-derived antigens and initiates T-cell-mediated inflammation. Patients with celiac disease may be more susceptible to lymphoproliferative diseases due to the ongoing activation of immunological pathways involving these genes, which may foster an environment that is pro-inflammatory.
- *STAT6 (NHL) and SH2B3 (Celiac Disease)* a possible overlap in cytokine-mediated immune responses is suggested by STAT6, which is linked to cytokine signaling in NHL, and SH2B3, which is involved in immune control in Celiac disease. In NHL, STAT6 is essential for IL-4 and IL-13 signaling, which supports Th2-mediated immune responses and aids in the survival and growth of lymphoma cells. Immune homeostasis depends on SH2B3, commonly referred to as LNK, which adversely regulates a number of cytokines receptor signaling pathways. Elevated immunological responses and heightened vulnerability to autoimmune illnesses, such as celiac disease, are linked to mutations in SH2B3. These genes’ deregulation may lead to abnormal immune cell proliferation and chronic inflammation, which could establish a connection between autoimmunity and lymphoproliferative disorders.
- *NOTCH2 (NHL) and TAGAP (Celiac Disease)* it’s possible that TAGAP, which activates T cells in celiac disease, and NOTCH2, which affects lymphocyte development in NHL, interact with T-cell-mediated immunity.

***NHL and SLE***

- *TRAF2 (NHL) and PTPN22 (SLE)* a shared immunological pathway is implied by the interaction between TRAF2 in NHL and PTPN22 in SLE, indicating that T-cell and NF-$\kappa$B signaling may be changed in these disorders. The NF-$\kappa$B pathway, which is essential for lymphoma cell survival, proliferation, and immunological responses, includes TRAF2 as a major mediator. Similarly, PTPN22 is closely linked to autoimmune diseases like SLE and is known to negatively regulate T-cell activation. Both autoimmunity and carcinogenesis depend on immune escape and chronic inflammation, which can result from dysregulation of both pathways. These results imply that by modifying immune responses and reestablishing normal signaling balance, focusing on this common pathway may have therapeutic potential for both NHL and SLE.
- *MLL3 (NHL) and FCGR2A (SLE)* a complex interaction between epigenetic regulation and immune responses is suggested by the correlation between MLL3’s function in chromatin remodeling in NHL and FCGR2A’s role in immune complex clearance in SLE. Through histone modifications, MLL3 plays a crucial function in controlling gene expression, which can affect the course of tumors. In contrast, FCGR2A plays a crucial role in removing immune complexes and reducing inflammation in SLE. When these genes are dysregulated, it can result in chronic inflammation and epigenetic modifications that encourage the growth of cancer or autoimmune reactions.
- *BCL6 (NHL) and BLK (SLE)* in NHL, germinal center formation is crucially regulated by BCL6, whereas BLK plays a role in B-cell receptor signaling in SLE. This points to a common genetic basis for the regulation and function of B cells. While BLK mutations are linked to decreased B-cell tolerance and auto-antibody production in SLE, BCL6 suppression can interfere with B-cell differentiation and encourage lymphomagenesis. These genes collectively point to a common pathway that could connect aberrant B-cell development to autoimmune and lymphoproliferative diseases.
- *MYC (NHL) and FCGR2B (SLE)* there may be a connection between immune response and dysregulation of cell proliferation, as suggested by MYC, a regulator of cell proliferation in NHL, and FCGR2B, an immune complex clearance factor in SLE. Aggressive lymphomas are often characterized by MYC overexpression, which promotes unchecked cell survival and proliferation. On the other hand, persistent inflammation and impaired immune complex clearance are associated with decreased expression or polymorphisms in FCGR2B in SLE. In autoimmune illnesses, these mechanisms might work together to encourage long-term immune activation, which would foster a milieu that is conducive to lymphomagenesis.
- *PTPN22 (NHL) and IRF5 (SLE)* PTPN22, involved in T-cell signaling in NHL, and IRF5, a regulator of interferon responses in SLE, highlight a potential intersection in T-cell regulation and immune signaling.
- *PTPN22 (NHL) and IRF5 (SLE)* a possible connection between T-cell control and immunological signaling is highlighted by PTPN22, which is implicated in T-cell signaling in NHL, and IRF5, which regulates interferon responses in SLE. Changes in T-cell receptor signaling linked to PTPN22 polymorphisms have the potential to exacerbate autoimmunity and reduce immunological tolerance. The development of auto-antibodies and chronic inflammation in SLE are also influenced by IRF5, a crucial transcription factor that enhances interferon signaling. When these genes are dysregulated, the immune system may become permanently activated, which raises the risk of lymphoma in people with autoimmune diseases.
- *CREBBP (NHL) and ITGAM (SLE)* a relationship between immune cell function and the regulation of gene expression is suggested by CREBBP, which influences chromatin remodeling and gene expression in NHL, and ITGAM, which is involved in immune cell adhesion in SLE. Histone acetylation disruption is commonly linked to CREBBP mutations in NHL, which results in aberrant gene expression and accelerates tumor growth. On the other hand, ITGAM is essential for leukocyte adhesion and migration, and polymorphisms in ITGAM have been connected to a higher risk of SLE by influencing the clearance of immune complexes. In the setting of autoimmune diseases, the combined deregulation of these genes may lead to chronic inflammation and compromised immune responses, which raises the risk of lymphoma development.

***NHL and Sjögren’s syndrome***

- *BCL2 (NHL) and IRF5 (Sjögren’s Syndrome)* a potential cross-pathway interaction is suggested by the connection between BCL2’s role in apoptotic control in NHL and IRF5’s role in the immunological response in Sjögren’s syndrome. In NHL, BCL2 over expression is frequently linked to diffuse large B-cell lymphoma (DLBCL) and follicular lymphoma, where it inhibits apoptosis to enhance cell survival. IRF5 polymorphisms have been connected to elevated production of pro-inflammatory cytokines and type I interferons in Sjögren’s syndrome, which has been implicated in the development of systemic characteristics and an elevated risk of lymphoma. Mucosa-associated lymphoid tissue (MALT) lymphoma is considerably more likely to occur in patients with Sjögren’s syndrome and high IRF5 expression, according to clinical research. This suggests that persistent activation of both pathways may encourage malignant transformation.
- *CDKN2A (NHL) and BAFF (Sjögren’s Syndrome)* in Sjögren’s syndrome, BAFF is necessary for B-cell survival and function, whereas CDKN2A regulates the cell cycle in NHL. This suggests a connection between altered B-cell physiology in autoimmune diseases and cell cycle dysregulation in lymphomas. Both p16INK4a and p14ARF, which are encoded by the tumor suppressor gene CDKN2A, are essential for controlling the cell cycle and halting unchecked growth. Increased tumor aggressiveness in NHL is linked to loss of CDKN2A function. On the other hand, increased BAFF levels in Sjögren’s syndrome encourage autoreactive B cell survival, which fuels persistent inflammation and the generation of autoantibodies. High BAFF expression-induced persistent B-cell activation may foster malignant transformation and raise the risk of B-cell lymphomas such mucosa-associated lymphoid tissue (MALT) lymphoma.
- *TNFRSF14 (NHL) and CXCR5 (Sjögren’s Syndrome)* in NHL, TNFRSF14 plays a role in lymphocyte activation. In Sjögren’s syndrome, CXCR5 is involved in the formation of germinal centers and lymphocyte migration. The connections between these genes might point to a common mechanism governing the behavior of lymphocytes and the dynamics of germinal centers. In germinal centers, TNFRSF14, often referred to as HVEM (herpesvirus entry mediator), controls the interactions between T and B cells. Increased lymphocyte survival and a higher incidence of follicular lymphoma are linked to its dysregulation in NHL. However, in Sjögren’s Syndrome, a chemokine receptor called CXCR5 directs B-cell migration to germinal centers in secondary lymphoid organs, promoting long-term immunological activation. The development of ectopic lymphoid structures in the salivary glands, a defining feature of Sjögren’s Syndrome and a recognized risk factor for the onset of lymphoma, has been connected to elevated CXCR5 expression.
- *CARD11 (NHL) and HLA-DQB1 (Sjögren’s Syndrome)* in NHL, CARD11 is essential for lymphocyte signaling and activation. In Sjögren’s syndrome, HLA-DQB1 is crucial for the presentation of antigens. This points to a possible connection between immune response activation and regulation. In NHL, constitutive activation of the NF-*kappa*B pathway due to mutations in CARD11 can promote unchecked lymphocyte proliferation. Similarly, by promoting the presentation of autoantigens and maintaining chronic inflammation, particular HLA-DQB1 alleles are closely linked to an elevated risk of developing autoimmune disorders, such as Sjögren’s Syndrome.

This study significantly advances the field with its thorough genetic analysis and innovative methodology. It illustrates how sophisticated computational techniques can be used to effectively decipher the intricate genetics of disease. Our results may direct future investigations and aid in the creation of focused treatments and diagnostics. Because of this variability, the suggested approach is more broadly applicable to complex illness study and is certain to capture a large range of genetic interactions. Future research, however, can concentrate on enlarging the dataset to incorporate more illnesses and genetic variants in order to confirm the model’s resilience and generalization. It may also be possible to increase the findings’ adaptability and applicability in various clinical contexts by combining this method with external datasets from various demographics.

## Conclusions

By utilizing the novel CB-BGWO approach, our study has effectively identified intricate genetic connections between Non-Hodgkin lymphomas (NHL), and a number of rheumatologic conditions, including Sjögren’s syndrome, Celiac disease, and SLE. The CB-BGWO strategy has been helpful in locating important genetic markers and clarifying their relationships, which has improved our comprehension of the complex nature of these diseases.

We have contributed significantly to our understanding of the pathogenesis of these diseases by identifying new genetic associations, which provide a more complete picture of their genetic foundations. These findings may have clinical implications for diagnosis, prognosis, and treatment in addition to adding to the corpus of scientific knowledge.

The effectiveness of this approach in processing and analyzing large volumes of genetic data is evidence of the value of using computational methods in biological research.

Particularly in the analysis of genomic data, the suggested Clustered-Based Binary Grey Wolf Optimizer (CB-BGWO) has shown good performance in feature selection and classification accuracy. There are still some practical issues, though, as with most optimization-based strategies. The initial clustering step may have an impact on the method’s efficiency and feature selection results in datasets with highly uneven feature distributions. Furthermore, even though the approach has been tested on a variety of datasets, additional improvement for computational efficiency may be necessary when applying it to larger-scale genomic datasets. To further improve scalability and resilience, future research can concentrate on combining parallel processing and adaptive clustering techniques. The CB-BGWO approach will also pave the way for additional investigations into the genetics of other complex diseases. Building on our findings, future studies could use this approach to find more genetic correlations and their implications. Furthermore, putting these genetic associations through experimental validation is essential to implementing these findings in clinical settings. Despite focusing on a specific case, we aimed to establish a high-performing model and prototype by creating a hybrid algorithm with various methods in this study.

## Data Availability

In accordance with the request of the readers of the publication, the data will be shared as applicable.
